# The impact of Cariprazine on Cholesterol, Glucose and Weight in patients affected by Schizophrenia: a systematic review and meta-analysis

**DOI:** 10.1192/j.eurpsy.2026.10425

**Published:** 2026-07-31

**Authors:** M. Pompili, M. Cifrodelli, A. V. Mammoliti, F. Formica, R. Iannazzo, R. McIntyre, I. Berardelli

**Affiliations:** 1 Department of Neurosciences, Mental Health and Sensory Organs, Faculty of Medicine and Psychology, Suicide Prevention Centre, Sant’Andrea Hospital, Sapienza University of Rome, Via di Grottarossa, 1035, 00189, Rome; 2 Department of Human Neursciences, Sapienza University of Rome, Rome; 3 Psychiatry Residency Training Program, Faculty of Medicine and Psychology, Sant’Andrea Hospital, Sapienza University of Rome, Via di Grottarossa, 1035, 00189, Rome, Italy; 4 Department of Psychiatry; 5 Department of Pharmacology and Toxicology, University of Toronto, Toronto, Ontario, Canada

## Abstract

**Introduction:**

Cariprazine is a second-generation antipsychotic approved for treating schizophrenia, acute manic or mixed episodes associated with bipolar I disorder and major depressive disorders. Antipsychotic treatment is often associated with metabolic alterations including weight gain, dyslipidemia, and increased risk of type 2 diabetes and cardiovascular disease

**Objectives:**

We performed a systematic review and metanalysis with the overarching aim of synthesizing study results that describe the effect of Cariprazine on glucose and lipid homeostasis as well as weight in persons living with schizophrenia.

**Methods:**

Three meta-analyses focused on patients with schizophrenia, evaluating changes in weight, cholesterol, and glucose for both Cariprazine 1.5 mg and 3 mg

**Results:**

This meta-analysis suggests that Cariprazine, although associated with modest weight gain, does not cause significant alterations in lipid and glycemic profiles.

**Conclusions:**

This meta-analysis confirm favorable metabolic profile of Cariprazine, compared to other atypical antipsychotics.

**Disclosure of Interest:**

None Declared

